# Endodontic implications of dental trauma: useful tips for primary dental care

**DOI:** 10.1038/s41415-025-8501-1

**Published:** 2025-04-11

**Authors:** Laura Gartshore, Tauseef Haq, Serpil Djemal

**Affiliations:** 41415410964001 School of Dentistry, Institute of Life Course and Medical Sciences, Faculty of Health and Life Sciences, University of Liverpool, UK; 41415410964002 Chair, Dental Trauma UK (dentaltrauma.co.uk), UK; Former Consultant in Restorative Dentistry, King´s College NHS Trust, UK

## Abstract

Dental trauma is the fifth most common condition affecting human beings worldwide. The dental team can expect to encounter traumatic dental injuries in everyday practice. Dental trauma guidelines guide practice and should be available to the dental team faced with an acute presentation of injury in a patient of any age. The management of dental trauma and the endodontic materials available for intervention of its complications are rapidly evolving. This paper provides an important update for the primary dental care team regarding the endodontic management of dental trauma.

## Key points

- Appropriate endodontic management of traumatic dental injuries has significant impact on prognosis and tooth survival.
- Dental trauma is a common condition - the fifth most common of all conditions worldwide - and those who experience trauma frequently report poor outcomes and significant impact on quality of life.
- Dental trauma management should remain at the forefront of practice for the entire team due to its prevalence, impact, complexity, and in recognition of how rapidly this area evolves in relation to evidence-based guidance and bioceramic materials.
- Commissioners should continue to recognise the prevalence, impact and interventions necessary to optimally manage the complications of dental trauma.

## Introduction

The most common five conditions affecting human beings are:

1. Caries
2. Tension headaches
3. Iron deficiency anaemia
4. Hearing loss
5. Traumatic dental injury (TDI).^1^^,^^2^

It has been estimated that almost one billion people in the world have experienced a TDI.^3^ TDIs are especially prevalent in children - up to a half experience TDIs to their primary teeth and one-third sustain trauma to their permanent teeth.^3^^,^^4^ The most common type of TDIs reported are uncomplicated crown fractures of the maxillary permanent incisors. Injuries to posterior teeth account for only 20% of all TDIs.^5^^,^^6^^,^^7^^,^^8^ Luxation injuries occur less frequently than fractures; however, when they do occur, complications are common.^3^ Only 10% of TDIs are described as complex, including avulsion, intrusion and crown-root fracture.^4^ Teeth may sustain a combination of injuries concurrently, with a negative, synergistic impact on prognosis.^5^ It has been estimated that by 50 years of age, 3.5% of adults have lost a tooth due to a TDI.^6^ There is strong evidence that tooth loss negatively impacts a person's quality of life.^7^^,^^8^

Appropriate management of the acute presentation of dental trauma has greater impact on prognosis than any other factor. Delay in treatment negatively impacts prognosis, and all those who might encounter TDIs are encouraged to keep up to date with knowledge in evolving therapies. This paper presents a summary of the complications that may arise following TDI, and considers the importance of appropriate endodontic management.

## Presentation of TDIs

Patients will often first seek treatment for dental trauma in a primary care setting.^9^ It is reported that some general dental practitioners do not feel confident in the management of TDIs due to not routinely encountering complex cases in practice.^10^^,^^11^^,^^12^ There are key features of management of TDIs that apply in all situations (Box 1). The International Association of Dental Traumatology (IADT) make guidelines for the evaluation and management of TDIs available on their website (https://iadt-dentaltrauma.org/) it is highly recommended that readers refer to these and have them available in practice. These guidelines are supported by excellent illustrations and are recommended as the go-to resource for the management of dental trauma. Furthermore, ToothSOS is a user-friendly app created by IADT and linked for download via this website. The app is designed for both professionals and patients and contains advice about the prevention of trauma and immediate and long-term management.

Patients may sustain TDIs in conjunction with other injuries and may present to hospital services.^13^ Many emergency department health professionals have received little or no formal training in the assessment and triage of TDIs and subsequently report a lack of confidence in their management.^14^^,^^15^ Inappropriate acute and endodontic management and advice may lead to undesirable consequences, including damage to a developing permanent tooth, pulpal necrosis leading to avoidable pain and dental abscess, or resorption and loss of a permanent tooth. There is a need to enhance dental trauma teaching for all emergency department health professionals who encounter TDIs to increase their confidence and enable them to triage and advise patients appropriately, and to request the support of hospital dental services in complex cases. Additionally, improved signposting for families to their most appropriate local dental service could in turn improve outcomes and experiences for children who experience a TDI.^11^^,^^16^

In the case of complex TDIs referred to specialist or secondary dental services, a multidisciplinary approach is often indicated, which may include input from paediatric dentists, restorative dentists, endodontists, prosthodontists, periodontists, orthodontists and oral surgeons, depending on the age and presenting complaint of the patient. This multidisciplinary approach is especially important in cases of complex trauma, where the endodontic management can be challenging and the prognosis uncertain.^17^^,^^18^^,^^19^^,^^20^

Box 1 A summary guide for management of TDIs in primary care The primary aims of the general dental practitioner when TDIs present are:

- Manage the acute trauma appropriate to positively impact prognosis
- Institute root canal treatment when indicated by guidelines
- Decide if any other injuries warrant root canal treatment
- Implement a follow-up protocol to monitor pulpal healing
- Refer to specialist or secondary care services when indicated

## Complications arising following TDIs

The incidence rate of complications following a TDI has been reported to range from 23-84%.^21^^,^^22^^,^^23^ Teeth which have sustained multiple episodes of a TDI are at a greater risk of complications (Box 2).^24^ Complication rates for TDIs have been evaluated for outcomes when the IADT guidelines have been followed, and it is worth noting that more favourable outcomes are observed in these cases compared to those when the guidelines are not followed.^22^ Patients who are informed of all potential outcomes from the time of injury may better appreciate the need for long-term follow-up and will be better prepared should symptoms arise.

Box 2 Summary of complications that may arise following a TDI

- Pulpal necrosis
- Pulp canal obliteration
- Root resorption
- Tooth discolouration
- Tooth loss

## Pulpal responses and reactions to TDIs

The pulpal response to a TDI varies dependent on the:

- Type of injury
- Amount of displacement of the tooth (and subsequently the neurovascular supply)
- Amount of crushing or stretching of the neurovascular bundle at the apex
- Degree of pulpal exposure
- Experience of previous trauma
- Experience of caries or previous restoration in the tooth
- Delay in presentation
- Patient compliance
- Socioeconomic group.^25^^,^^26^

The pulp can respond in a variety of ways following a TDI. The following might be expected depending on the above factors:

- Full recovery and healing of the pulp
- Pulp revascularisation, particularly in immature teeth^26^^,^^27^
- Tertiary dentine formation
- Pulp necrosis
- Pulp canal obliteration.

Pulpal necrosis is the most frequent complication occurring in approximately 34% of teeth that have sustained injury (Table 1).^24^

**Table 1:** Injuries highly likely to be associated with pulp necrosis

**Open apex tooth**	**Closed apex tooth**
Intrusion + crown fracture	Extrusion + crown fracture
Avulsion + crown fracture	Lateral luxation + crown fracture
	Intrusion
	Avulsion

The clinical signs and symptoms of pulp necrosis to be aware of are:

- Pain
- Swelling
- Presence of a sinus tract/localised swelling over the root of the tooth
- Discolouration
- Tenderness to palpation
- Tenderness to percussion.

The radiographic signs of pulp necrosis to be aware of following a TDI are:

- Increase in the periodontal ligament space
- Presence of an apical radiolucency.

It is common practice to record two or more signs or symptoms of pulp necrosis in order to make a diagnosis. While the use of sensibility tests (eg a cold spray such as Frost and electric pulp testers) may be useful as a baseline, they do not represent an accurate reflection of the state of the pulp following acute trauma, nor in young children with immature apices. They are, however, useful to monitor possible disease progression and to corroborate the reported clinical and radiographic findings. Therefore, the results of testing should be recorded at every appointment; although, they should not be considered a strong indicator of pulp death following a TDI.

## Management of the pulp following TDIs

In most cases, the pulp should be given a chance to heal and survive. There are, however, some injuries where endodontic treatment is indicated as part of the acute management. These include avulsion of mature permanent teeth, immature permanent teeth with an extended extra-oral dry time (>60 mins), intruded and severely luxated teeth, and complicated crown fractures where it has not been possible to achieve haemostasis when a pulpotomy is attempted. If root canal treatment is indicated soon after injury, this should be commenced without delay. Additionally, there are a few scenarios where endodontic treatment is prescribed to facilitate retention of a coronal restoration. This is usually associated with cervical-third root fractures and crown-root fractures that, by definition, have a poorer long-term prognosis. All other patients who have sustained a TDI should be placed onto a follow-up pathway to monitor signs and symptoms.

Pulp necrosis can be sterile (immediately following an avulsion) or infected.^26^ An infected pulp results from bacterial ingress which may be via infractions within the enamel/dentine, microleakage around existing restorations and through the root dentine. When there are bacteria in the root canal, this can damage the cementum and lead to inflammatory root resorption.^28^ It is important, therefore, to monitor traumatised teeth to allow for early detection and treatment of pulpal necrosis. Generally, apical resorption is due to bacterial infection within the root canal and in cases of intrusion where there is crushing of the apical tissues.

## Pulp canal obliteration

Pulp canal obliteration (PCO) occurs due to the accelerated deposition of hard tissue within the pulp chamber and is evident in 3.5-64% of cases of TDI.^29^^,^^30^^,^^31^^,^^32^ Active deposition of tertiary dentine within the tooth aims to narrow the pulp chamber and reduce the risk of pulpal necrosis. PCO can cause discolouration which may cause aesthetic concerns and there is a risk of pulpal necrosis in 7-27% of cases.^31^ Prophylactic endodontic treatment is not advised in these discoloured teeth unless there are clear symptoms of periapical periodontitis.^33^ PCO and subsequent necrosis can prove challenging for the treating clinician to manage due to the reduced pulp space. Cone beam computed tomography (CBCT) investigation can be very helpful and referral to a specialist is often advised.

## Root resorption

**Fig. 1:**
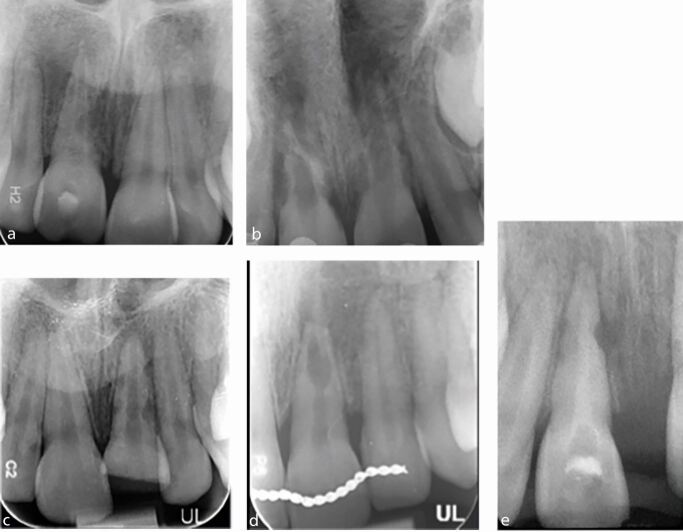
a, b, c, d, e) Series of various periapical radiographs of traumatised teeth experiencing infection-related and replacement-related resorption

Root resorption is defined as the loss of dental hard tissue as a result of osteoclastic cell action.^34^ Several types of root resorption can occur following TDIs, including infection-related resorption, replacement-related resorption, cervical root resorption and surface resorption. Root resorption is being increasingly diagnosed by clinicians, most likely due to improved access to advanced means of diagnosis, such as CBCT, which is more likely to detect resorption than plain film radiographs.^35^^,^^36^ CBCT provides a comprehensive 3D evaluation of the site and is consistently useful for guiding a surgical approach and minimising iatrogenic damage, as well as identifying resorption. The most common type of root resorption following TDI is infection-related, followed by replacement-related.^37^ Infection-related resorption presents radiographically as a radiolucent lesion/s, whereas replacement resorption will present a more radio-opaque appearance where bony infill is apparent in resorptive defects and in direct contact with the root surface (Fig. 1).

Resorption can occur on the internal and external aspects of the root surface (Table 2). In infection-related resorption, damage to the protective superficial layer of cementum or predentine must occur in the presence of bacteria.^38^ Once it starts, the resorptive process will progress for as long as the root canal remains infected, which can result in loss of the whole root and subsequently tooth loss, and this can be rapid in children. Importantly, appropriate endodontic treatment to remove the necrotic pulp and to achieve a root canal rendered free from the presence of bacteria (by dressing with non-setting calcium hydroxide) can arrest the progression of the infection-related root resorption.^28^

**Table 2:** Injuries highly likely to be associated with pulp necrosis

**Internal resorption**	**External resorption**
Commences within pulp or in the dentine of the root canal walls	Commences within the cementum and/or dentine
Progresses outwards towards cementum	Progresses inwards towards pulp
If not treated, it can result in communication with the periodontal ligament and surrounding bone	If not treated, it can reach the pulp, resulting in communication between the pulp and the surrounding bone
(Infection related, replacement related, surface)	(Infection related, replacement related, surface, orthodontic, idiopathic)

Replacement-related resorption is the pathological union of cementum or dentine of a tooth to the surrounding alveolar bone, commonly referred to as ankylosis.^39^ After ankylosis, a tooth becomes involved in the bone remodelling cycle and can be broken down by osteoclasts and replaced with bone by osteoblasts. Replacement-related resorption is a particular complication of teeth that have been avulsed and reimplanted, or that have suffered a severe intrusion injury.^40^ Teeth experiencing replacement-related resorption may have high percussion tone, reduced or no physiological mobility, loss of periodontal ligament space, and impaired discrimination between the lamina dura and root dentine. Failure of attempted orthodontic movement is also diagnostic.

**Fig. 2:**
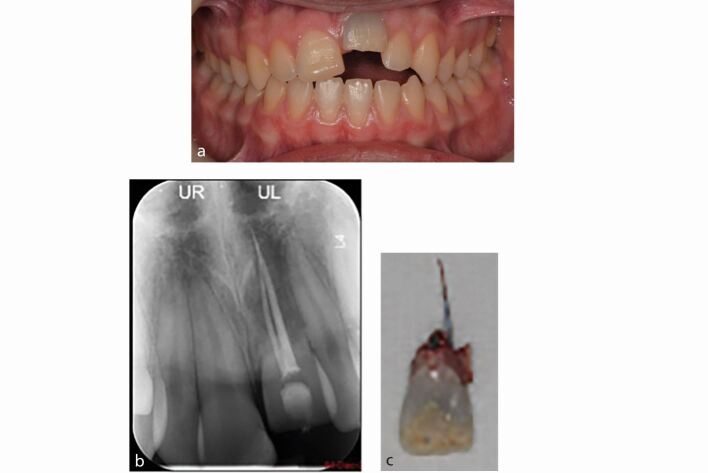
a, b, c) Late presentation of replacement-related resorption and infraocclusion of the 21 in a growing child during the COVID-19 pandemic

**Fig. 3:**
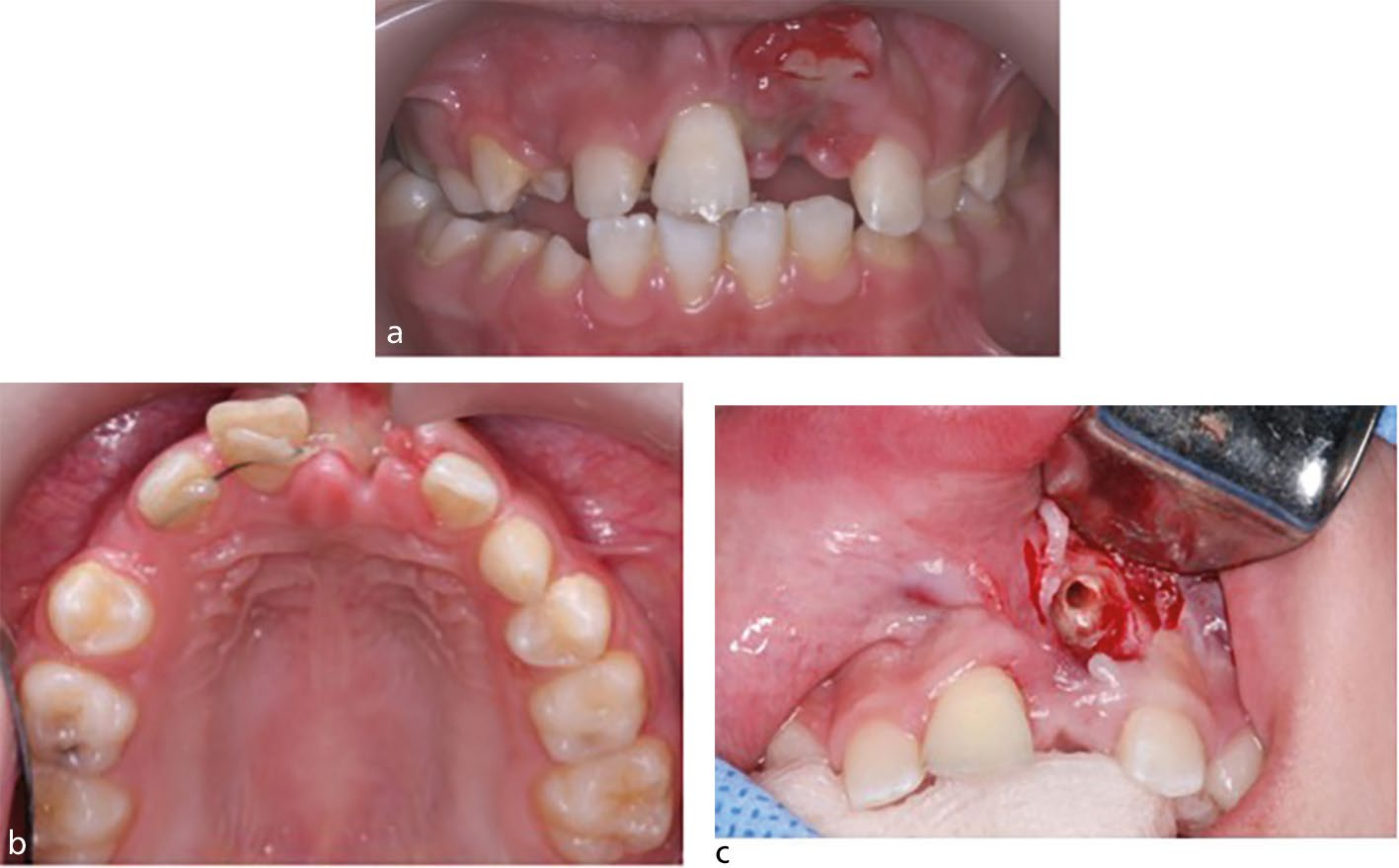
a, b, c) Dental trauma managed outside of guidelines following avulsion of the 11 and severe intrusion of the 21 in a growing child, leading to replacement-related resorption and the eventual decoronation of both teeth

If less than 20% of the tooth surface is involved, the ankylosis may only be a transient; however, if it is more established, there is no effective treatment.^41^ In a growing child, this situation leads to infraocclusion of the affected tooth, and arrest of vertical growth height of the alveolar ridge leading to an alveolar defect that may be difficult to restore prosthetically (Fig. 2). Children may present with tilting of the adjacent teeth due to continued growth and development of the adjacent teeth and alveolus compared to the ankylosed tooth and its related alveolus. This is a particular risk from the age of ten years until completion of the early teenage growth spurt. These cases should be referred for urgent specialist management. Management options are limited and often involve decoronation (to preserve the alveolar bone until adulthood when advanced restorative options can be considered) or tooth loss (extraction may be destructive and leave a residual vertical bony defect) (Fig. 3).

Cervical root resorption is an aggressive form of external root resorption, which is relatively uncommon. It presents as a localised area of resorption that occurs in the zone of connective tissue attachment. The dental pulp plays no part in this type of resorption and is mostly normal unless the resorption has progressed to a very late stage.^42^

Surface resorption is a self-limiting phenomenon. It may be noted as an altered root contour involving the cementum and dentine, and no intervention is necessary.^43^

## Tooth discolouration

Teeth that have sustained a TDI can discolour as a result of injury or intervention. Tooth discolouration can impact a person's self-confidence, perceived physical attractiveness and employment prospects, and may lead to negative social judgement between children.^44^^,^^45^ The colour of a traumatised tooth will be impacted by the pathological process occurring within the tooth. Pulpal necrosis can result in a grey discolouration occurring due to haemolysis of red blood cells and pulpal remnants within the tooth, which can penetrate the dentinal tubules.^46^^,^^47^ Discolouration is often noted at 4-24 months post-injury.^48^ PCO can result in a yellow hue due to the extra deposition of dentine within the tooth.^31^ Cervical root resorption is noted as a pinkish hue developing in the cervical region due to the presence of vascular tissue.^34^ Tooth discolouration following a TDI may also be transient due to temporary disruption of the blood supply to the tooth, or permanent, and may follow intervention eg resulting from bismuth oxide in mineral trioxide aggregate.^43^^,^^49^

## Management of immature teeth following a TDI

**Fig. 4:**
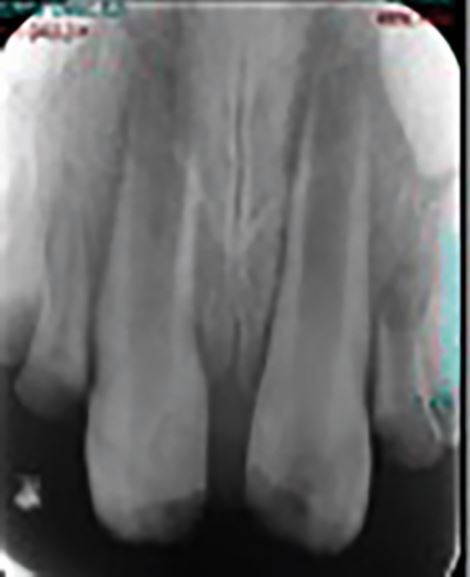
Teeth that are traumatised and subsequently experience pulpal necrosis and apexogenesis in young children might have very immature roots that are susceptible to fracture. Regenerative endodontic procedures are evolving to provide a possible improved prognosis for these cases in the future

Recovery and healing of the pulp (revascularisation) is more likely in immature teeth with open apices. Pulp necrosis in immature permanent teeth can result in cessation of root development with a resultant weakened root, apical inflammation, inflammatory root resorption, and apical bone loss. Immature roots are fragile and susceptible to root fracture, even to mild forces in the future. Recent developments and research in paediatric dentistry and endodontics have aimed to progress the potential of regenerative endodontic procedures.^50^^,^^51^^,^^52^ There is much to learn about the nature of the vital tissues that might be regenerated, the optimal tissue scaffold that might be required, and the ideal disinfection strategy to support success. It is largely agreed that regenerative endodontic procedures may develop to offer an improved prognosis where there has been careful case selection of immature traumatised teeth. Such cases include patients aged 7-9 years, when the root walls may be especially thin and susceptible to fracture, even in the presence of apical root end closure procedures with hydraulic calcium silicate cements (Fig. 4).^53^ The management of the child who has experienced complex dental trauma is often best guided by a specialist in paediatric dentistry, with the primary dental care practitioner providing invaluable support.

## Use of hydraulic calcium silicate cements (bioceramics) in the management of TDIs

Hydraulic calcium silicate cements are products with several properties which make them beneficial for the management of necrotic teeth following a TDI. These materials are indicated to be used for the following procedures:

- Pulp capping and pulpotomy - in appropriate cases of pulp exposure, a biocompatible bioceramic with excellent sealing properties can be used with the aim of maintaining vitality and promoting apexogeneis^54^^,^^55^
- Apexification in immature teeth with open apices and management of resorptive defects - hydraulic calcium silicate cements are commonly used because of their excellent sealing properties and ability to stimulate periapical healing and root end closure. The materials are not sensitive to moisture during setting, making them suitable for procedures where moisture control can be challenging^56^
- Regenerative endodontic procedures - the sealing material is in direct contact with a blood clot so needs to be able to tolerate moisture and be biocompatible, bioactive and antimicrobial. Hydraulic calcium silicate cements are bioactive as they stimulate the deposition of hard tissue and promote dentine bridge formation.^56^ Silicon ions induce osteoblast proliferation and gene expression by involvement in metabolism, collagen synthesis, bone mineralisation, and connective tissue crosslinking^57^
- Endodontic treatment - hydraulic calcium silicate-based sealers can be used for their ability to form an antibacterial, hermetic seal with excellent biocompatibility and high pH. High alkalinity inhibits the growth of microorganisms and disinfects dentine.^58^ They can be used with the single-cone technique which is efficient for the operator.

## Conclusion

Dental trauma is common and commonly presents to primary dental care. Appropriate endodontic management of dental trauma has signficiant impact on prognosis and tooth survival. Multidisciplinary working, including the primary dental care team, is invaluable for the optimal recognition, diagnosis, referral and the endodontic management of TDIs.
